# Sensor to Electronics Applications of Graphene Oxide through AZO Grafting

**DOI:** 10.3390/nano13050846

**Published:** 2023-02-24

**Authors:** Suresh Sagadevan, Md Zillur Rahman, Estelle Léonard, Dusan Losic, Volker Hessel

**Affiliations:** 1 Nanotechnology & Catalysis Research Centre, University of Malaya, Kuala Lumpur 50603, Malaysia; 2 Department of Mechanical Engineering, Ahsanullah University of Science and Technology, Dhaka 1208, Bangladesh; 3 Research Center Royallieu, TIMR (Integrated Transformations of Renewable Matter), ESCOM, University de Technologie de Compiegne, CS 60 319, CEDEX, 60 203 Compiegne, France; 4 School of Chemical Engineering, The University of Adelaide, Adelaide, SA 5005, Australia; 5 The ARC Graphene Research Hub, School of Chemical Engineering and Advanced Materials, The University of Adelaide, Adelaide, SA 5005, Australia; 6 School of Engineering, University of Warwick, Library Rd, Coventry CV4 7AL, UK

**Keywords:** azobenzene, graphene oxide, synthesis, AZO-GO, AZO-RGO, applications

## Abstract

Graphene is a two-dimensional (2D) material with a single atomic crystal structure of carbon that has the potential to create next-generation devices for photonic, optoelectronic, thermoelectric, sensing, wearable electronics, etc., owing to its excellent electron mobility, large surface-to-volume ratio, adjustable optics, and high mechanical strength. In contrast, owing to their light-induced conformations, fast response, photochemical stability, and surface-relief structures, azobenzene (AZO) polymers have been used as temperature sensors and photo-switchable molecules and are recognized as excellent candidates for a new generation of light-controllable molecular electronics. They can withstand trans-cis isomerization by conducting light irradiation or heating but have poor photon lifetime and energy density and are prone to agglomeration even at mild doping levels, reducing their optical sensitivity. Graphene derivatives, including graphene oxide (GO) and reduced graphene oxide (RGO), are an excellent platform that, combined with AZO-based polymers, could generate a new type of hybrid structure with interesting properties of ordered molecules. AZO derivatives may modify the energy density, optical responsiveness, and photon storage capacity, potentially preventing aggregation and strengthening the AZO complexes. They are potential candidates for sensors, photocatalysts, photodetectors, photocurrent switching, and other optical applications. This review aimed to provide an overview of the recent progress in graphene-related 2D materials (Gr2MS) and AZO polymer AZO-GO/RGO hybrid structures and their synthesis and applications. The review concludes with remarks based on the findings of this study.

## 1. Introduction

Materials and nanostructures are the backbones of modern society [1]. Graphene oxide has a distinct two-dimensional (2D) carbon structure and highly tunable electronic properties [2,3]. Owing to these properties, graphene has significant fabrication potential with surface functionality [4]. Graphene-based nanomaterials demonstrate good antibacterial properties; however, their interplane solid interactions tend to aggregate, limiting their surface region and methods of activity [5]. Graphene is an excellent material for storing and accepting electrons generated by photons. Because of its high specific surface area and electrical conductivity, it can promote photo-induced charge separation and improve interfacial charge transport while extending the lifetime of photogenerated electron/hole pairs [6,7,8]. Graphene with a 2D structure consists of a hexagonal lattice structure with monolayer sp^2^ hybridization carbon atoms with significant catalytic performance in photocatalysis owing to its superior electron capturing and transport conductivity, large specific surface area, and superior interaction with other catalyst particles [9]. It has outstanding properties, including good thermal conductivity of approximately 5000 W m^−1^ K^−1^, 97.7% transparency to visible light, large carrier mobility (200,000 cm^2^ V^−1^ s ^−1^), large surface area, ambipolar electric field effect, and quantum Hall effect at room temperature [10,11,12]. Graphene derivatives (GDs) have given rise to a new class of multifunctional nanomaterials with distinct properties.

The GO and RGO are used as supporting platforms for biological binding processes due to their ease of functionalization, non-covalent binding, and variable hydrophobicity [13,14,15]. Biosensors based on various detection principles have been extensively studied for graphene-based biomedical applications, including harnessing graphene’s unique electronic properties in different biosensors for detecting a diverse group of biomolecules, including proteins, enzymes, and hormones [16,17,18,19,20,21,22,23,24,25]. Because graphene has a higher capacity to adsorb biomolecules than typical Au or Ag surfaces, it has widely been employed as a sensitivity enhancer in optical biosensors such as fiber-optic surface plasmon resonance detectors [26]. Gas sensors are used in various applications, including security, food safety, environmental monitoring, indoor air quality monitoring, and personal healthcare [27,28,29,30]. Two-dimensional (2D) nanostructured materials have received considerable attention over the last decade due to their unique chemical and physical properties. Because of their large surface-to-volume ratio, high surface sensitivity, and excellent semiconducting properties, they show promising potential for use in gas-sensing devices. Theoretical studies [31,32] showed that the adsorption of various gases onto graphene results in inconsistent doping states. The symbiotic effects of multiple components increase the sensitivity of pure graphene to gas molecules when blended with other functional materials. Carbon-polymer composite-based chemiresistors exhibit high stability, long lifetime, tunable selectivity, reversibility, and reproducibility [33]. Semiconductors [34], carbon materials [35], and organic/inorganic composites [36,37,38] have been used as sensing materials; however, they work on different mechanisms and principles. The two-dimensional structures of GDs provide an excellent platform for assembling AZO molecules in a close-packed order. Moreover, smart materials are being increasingly reported and used in various applications. However, the azobenzenic family is the most widely used because it is simple to synthesize, resistant to mechanical exposure, and has a good aging capacity under light illumination for photoisomerization. Therefore, this review focused on the most recent advancements in graphene-related 2D material (Gr2MS) and AZO hybrid structures and their applications.

### 1.1. Graphene-Related 2D Material (Gr2Ms) Foundation to All Carbon Materials

**Figure 1:**
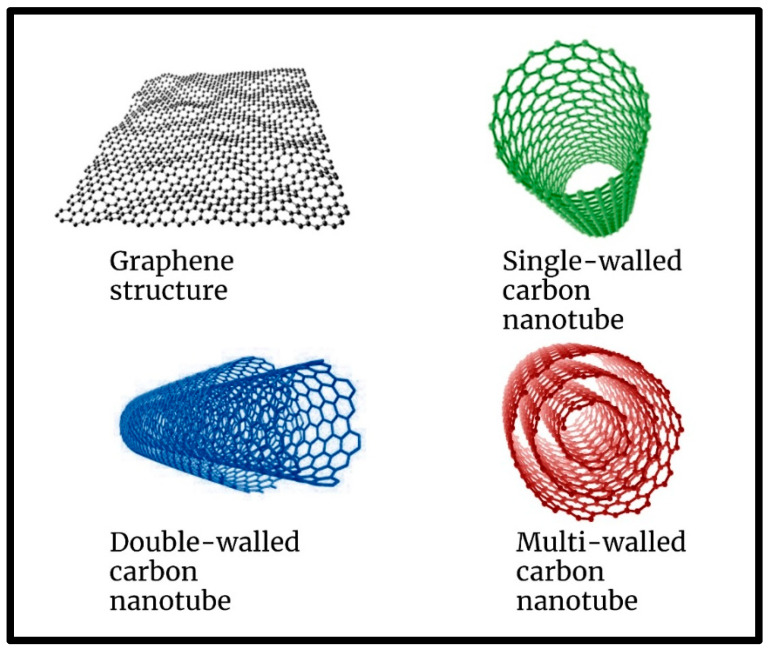
Graphene structure of single, double, and multi-walled carbon nanotubes.

Among different materials, carbon-based materials play an important role in civilization [39]. Carbon, the most important element in the periodic table, is particularly important. Diamond and graphite are the most well-known forms of carbon; however, it has long been recognized that carbon can be found in nature in a variety of compounds, including various types of amorphous carbon, organic molecules, and biomolecules [40,41]. The multiform nature of carbon is due to the peculiar characteristics of its unique electronic structure, which forms different hybrids, namely sp^1^, sp^2^, and sp^3^ [39]. The different orientations of the orbitals in these hybrids result in structures with vastly different properties [39]. Carbon has a wide spectrum of compounds and allotropic forms with different structures in 0D, 1D, 2D, and 3D [42]. Carbon at the nanoscale with sp^2^ carbons (e.g., nanotubes, fullerenes, nanofibers, nanocones, nanodiamonds, graphene, and graphene nanoribbons) with relatively large surface areas exhibits novel properties and is used in different industrial sectors [42,43,44,45,46,47]. The functionalization of these carbon nanomaterials affects different properties, such as their biocompatibility and toxicity toward the environment and living organisms [48,49,50,51,52]. Graphene is usually divided into a few layers, such as single-wall, double-wall, and multi-wall carbon nanotubes, as shown in Figure 1. These highly adaptable carbon backbones allow for easy functionalization and integration into a variety of applications [53]. Recent work [54] on the fabrication of 30-inch multilayer graphene sheets and their transport in roll-to-roll fabrication shows that they can easily be fabricated for large-scale use. The first mechanical extraction of graphene from graphite using a simple Scotch tape approach is described in refs. [55,56]. Graphene is the basic component of graphite, which consists of graphene layers stacked on top of each other with an interlayer spacing of 3.34 angstroms [57]. CNTs were first discovered and described in 1952 and then in 1976 [58]. In 1991, Iijima [59] was the first to report the formation of multi-walled carbon nanotubes (MWCNTs) because of the random nature of arc evaporation of C60 and other fullerenes. CNTs (carbon nanotubes) are small carbon tubes with diameters measured in nanometers. Carbon nanotubes are single-walled carbon nanotubes (SWCNTs) with dimensions in the manometer range. Single-wall carbon nanotubes are carbon allotropes that fall between fullerene cages and flat graphene [59]. CNTs, with a diameter of 1 nm and a length of a few nanometers to microns, are the most important form of carbon. CNTs are configurationally equivalent to a 2D graphene sheet rolled into a tube. As a result of oxidation, carboxyl groups are added to the surface of the CNT, which are useful for further modification. This facilitates the covalent coupling of molecules through the formation of amide and ester bonds. As a result, a wide range of functional moieties was created. The van der Waals interactions between CNTs are reduced in the presence of carboxyl groups, increasing the separation of the nanotubes. Suitable group attachments increase the solubility in organic or aqueous solvents, with the possibility of further modifications.

### 1.2. AZO Dyes

**Figure 2:**
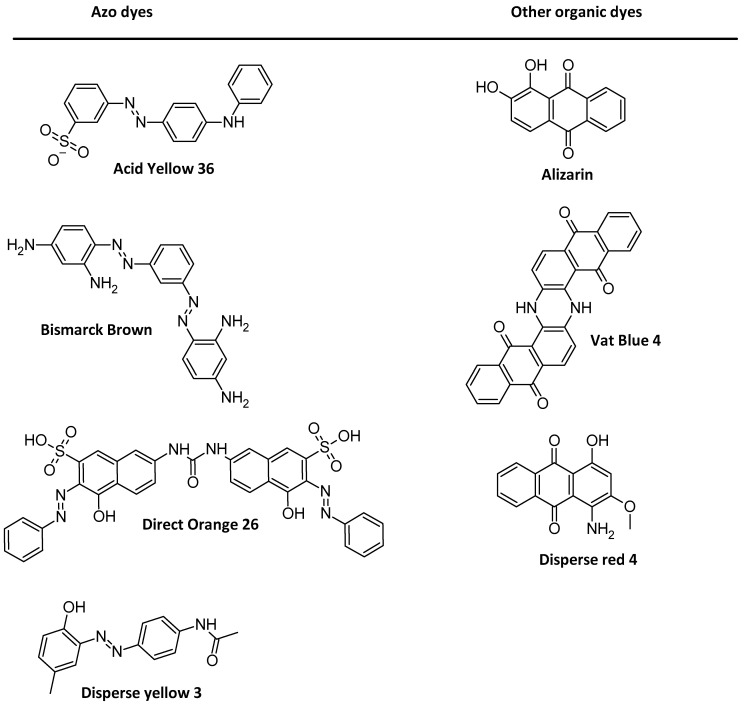
Examples of the most commonly used dyes in the textile domain.

Organic dyes are one of the biggest families in the synthetic organic domain. For example, in the textile domain, the most common dyes can be classified in two ways: (i) based on their application characteristics or (ii) based on their main chemical structure [60,61,62]. These structures can often be found in azobenzene dyes, as seen in these examples of most used textile dyes (Figure 2). Antracenedione moieties are frequently found in non-azo dyes, while ionic or non-ionic compounds may be present in azo dyes. If these latter molecules can be part of the most worldwide pollution, when strictly used, they can be fundamental for many applications such as electronics or nanomotors.

**Figure 3:**
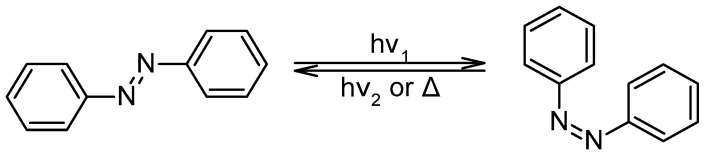
Azobenzenes and their switch capability.

The azobenzene family is the photochromic molecule capable of changing shape and polarity upon light irradiation. Azobenzene is a molecule able to change conformation from trans to cis upon light (Figure 3) and reversibly under another wavelength or heat [63,64,65]. The variation of HLB (hydrophilic–lipophilic balance) and polarizability of the trans-to-cis molecules are of great interest for the reached applications. Also, the ease of synthesis and the physical capabilities of azobenzene and derivatives led authors to study functionalized graphene oxide (GO) with azo benzenes (AZO). As a result, the functional synergy suggests that incorporating the AZO chromophore into the graphene sheets significantly improves photocurrent switching.

## 2. General Review of Graphene-Based Materials and GO-AZO Syntheses

This section discusses existing graphene-based materials that can serve as the basis for functionalization and GO syntheses available in the literature. Various functionalization methods for graphene and the azobenzenic functionalization of GO are also discussed.

### 2.1. Existing Graphene-Based Materials Available for Hybridization

**Figure 4:**
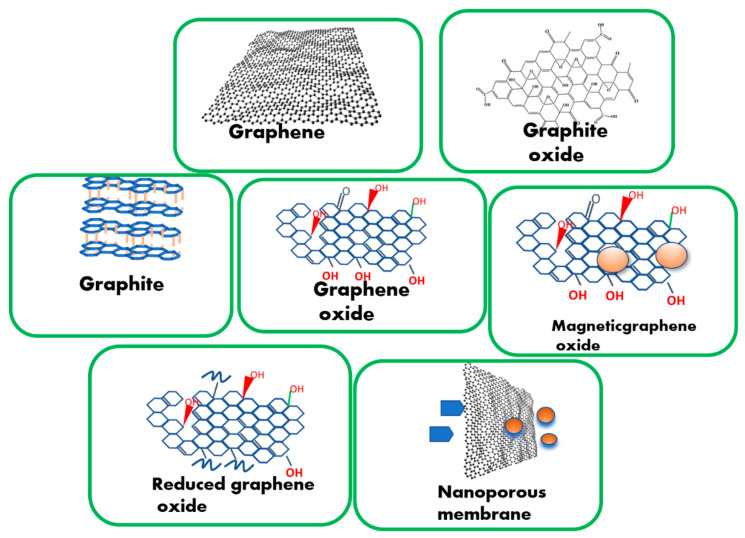
Schematic representation of graphene-based materials (Gr2Ms) [66].

Graphene oxide, which has better oxidative characteristics than graphene, or nanoporous graphene (NPG) could be employed instead of graphene alone [66,67]. Because of its vast specific surface area, high optical transmittance, and outstanding electrical characteristics, graphene is frequently utilized as a catalyst and promoter. A mechanical exfoliation process for single and multi-layer graphene was created from mineral graphites. However, graphene is considered a zero-band gap semiconductor, indicating that its band structure is linearly dispersed and the charge carriers act as massless. Dirac-fermions in a particular k-point in the first Brillouin zone [68]. Because graphene has a zero-band gap, it absorbs light in various spectra ranging from infrared to ultraviolet, allowing it to be used in electro-optical systems/devices [69]. Because of the conduction and valence band overlap, graphene behaves as a zero-gap semiconductor with large carrier mobility at ~106 ms^−1^ (relativistic speed) [70]. Graphene is considered a gapless material because the conduction and valence band are symmetrical and meet at the same Dirac point [71]. Graphene is a unique 2D material with one-atom-thick carbon atom layers, absorbing 2.3% of the total incident light [72]. Because of its superior electrical characteristics, graphene may efficiently increase charge separation and inhibit the recombination of excited carriers produced by photocatalysts [73]. Graphene and modified graphene are introduced in several fields of catalysis because of their excellent characteristics [74]. A schematic representation of graphene-based materials is illustrated in Figure 4 [66].

**Figure 5:**
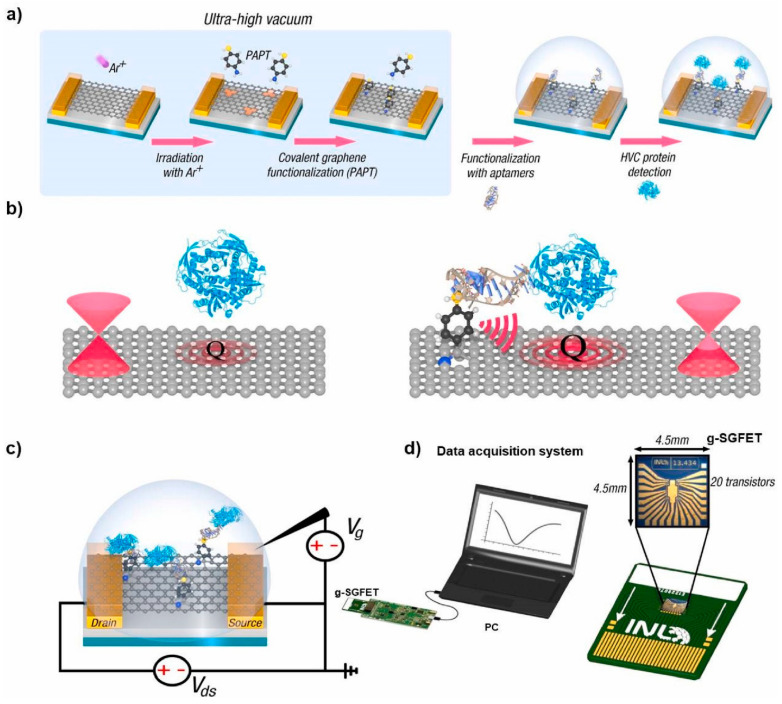
(**a**) Protocol to develop a covalent g-SGFET aptasensor, (**b**) molecular-antenna effect, (**c**) g-SGFET functioning scheme, and (**d**) real image and dimensions of the g-SGFET wire-bonded to a printed circuit board (PCB) inserted into an electronic platform that can communicate with a computer where the measured data are displayed [76].

The functionalization of graphene and graphene derivatives has increased the number of potential applications of graphene-based materials. Covalent functionalization, non-covalent functionalization, substitutional doping of graphene, and hybridization with nanoparticles, nanowires, and other materials have been classified as functionalization modes based on the methods and materials used [75]. These various functionalization methods provide new ways to expand the current applications of graphene, such as bioimaging and bandgap opening, for use in electronics [75]. Graphene field-effect transistor-based biosensors have also emerged as promising tools for detecting a wide range of analytes. However, the functionalization protocol displays a significant impact on their performance. Palacio et al. [76] developed an ultrasensitive aptamer-based biosensor (aptasensor) capable of detecting hepatitis C virus core protein using a controlled in-vacuum physical method for the covalent functionalization of graphene. These devices are highly specific and robust and can detect viral proteins at the attomolar level in human blood plasma protocol to develop a covalent g-SGFET aptasensor, as shown in Figure 5 [76].

### 2.2. GO Synthesis

There are two approaches:top–down and bottom–up, to synthesize GO. In thetop–down approach, physical methods such as mechanical/ball milling, sputtering, laser ablation, etc., are used. In chemical methods, there are two approaches such as chemical and green routes. It is necessary to define graphene composites before proceeding with the analysis. Graphene composites are any graphene-based materials that have been changed (grafting with reactive groups, functionalization with polymers, complexes with other sources, etc.). The GO is the most well-known graphene composite material, produced via the chemical exfoliation of graphite. Graphene has been severely oxidized and contains a range of oxygen functions. Many theories [77,78] have been proposed in the past to establish the precise chemical structure of GO. This is due to the material’s complexity (including sample-to-sample variability) and its non-stoichiometric amorphous berthollide atomic composition [79]. The three preparation methods for GO are (i) Brodies [80], (ii) Staudenmaier [81], and (iii) Hummer [82]. The most significant aspect of these methods is the chemical exfoliation of graphite with an oxidizing agent in the presence of mineral acid. Two methods (Brodies and Staudenmaiers) use a mixture of KClO_4_ and HNO_3_ to oxidize graphite. Hummer’s approach combines graphite, potassium permanganate, and H_2_SO_4_ in a solution. The conjugation of stacked graphene sheets is broken down into nanoscale sp^2^ graphitic domains surrounded by extremely disordered oxidized domains (sp^3^ C/C) and carbon vacancy defects when graphite is oxidized [83]. The phenolic, hydroxyl, and epoxy groups on the basal plane and the carboxylic acid groups on the margins cause the GO films to peel off easily and form a persistent tan-colored monolayer suspension in water [84].

### 2.3. Functionalization of GR2Ms

**Figure 6:**
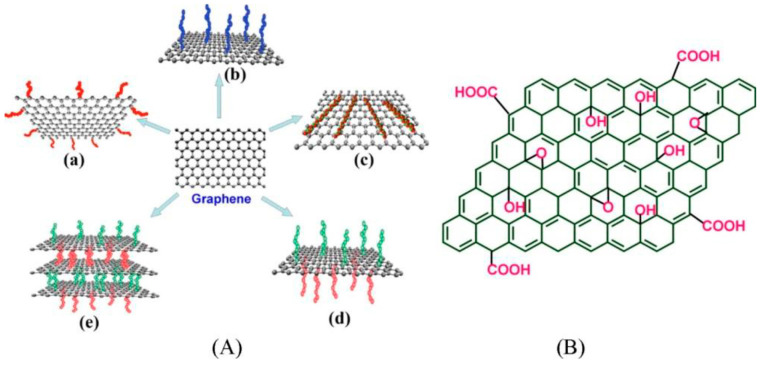
(**A**) Functionalization possibilities for graphene: (**a**) edge functionalization, (**b**) basal plane functionalization, (**c**) non-covalent adsorption on the basal plane, (**d**) asymmetrical functionalization of the basal plane, and (**e**) self-assembling of functionalized graphene sheets, and (**B**) Chemical structure of GO [91].

Hummers and Offeman [82] developed a technique for producing GO that involved strong chemical oxidation of graphite, followed by sonication for exfoliation of the obtained GO. GO is very hydrophilic due to the distribution of epoxide and hydroxyl groups on the basal planes and the positioning of carbonyl and carboxyl groups at the edges. Because the GO structure contains oxygen-rich functional groups, it is easily exfoliated in water and distributed into single-layer sheets, becoming stable in this manner. GO (or graphite oxide in some cases) is one of the most promising graphene derivatives. According to Wang et al. [85], the main routes of GO production are chemical oxidation and exfoliation of graphite using the Brodie, Staudenmaier, or Hummers method or some variations in these methods. Brodie observed that only graphitizable carbons with graphitic structural regions could be formed by the oxidizing combination (KClO_4_ with fuming HNO_3_) [86]. The formation of GO was then described by Staudenmaier when heating graphite with H_2_SO_4_, HNO_3,_ and KClO_4_ [87]. Later, Hummers and Offeman [82] developed a simple method to prepare GO using H_2_SO_4_ and KMnO_4_. There are two ways to prepare RGO: chemical reduction and annealing at a high temperature. The most effective thermal annealing method [88] is thermal deoxygenation of GO (see Figure 6), which is assisted by temperature elevation and eliminates O-based moieties such as OH [89,90]. However, this process is energy-intensive, and the degree of oxidation is difficult to control. Chemical reduction requires reducing agents such as metal hydrides, hydrazine, hydroiodic acid, and a narrow temperature range, and targeting these functional groups is difficult. The superior properties of nanofiltration membranes based on RGO over GO have led to several studies. Graphene, with a π-rich electronic structure, has a two-dimensional honeycomb lattice of sp^2^ hybridized carbon atoms. Graphene can easily be converted to GO and RGO. G is hydrophobic, whereas GO is hydrophilic. As a result, GO is easily dispersible in water. GO contains aliphatic (sp^3^) and aromatic (sp^2^) domains, increasing surface interactions. A range of approaches for surface modification of graphene has been developed in response to the increased interest in employing graphene as a reinforcing filler in polymer matrices to construct multifunctional materials. The filler-matrix bonding interaction considerably impacts the final composite properties. Most dispersion processes result in non-covalent composites in which the polymer matrix and filler connect via relatively weak dispersion forces. To enhance stronger interfacial interaction, researchers are increasingly focusing on creating covalent connections between graphene-based filler and the supporting polymer. Graphene becomes best functionalized via sp^2^ stacking complexation or by inserting particular moieties on edge or basal planes (Figure 6) due to the abundance of sp^2^-conjugated carbons [91].

### 2.4. AZO-Functionalization of Gr2Ms GO

**Figure 7:**
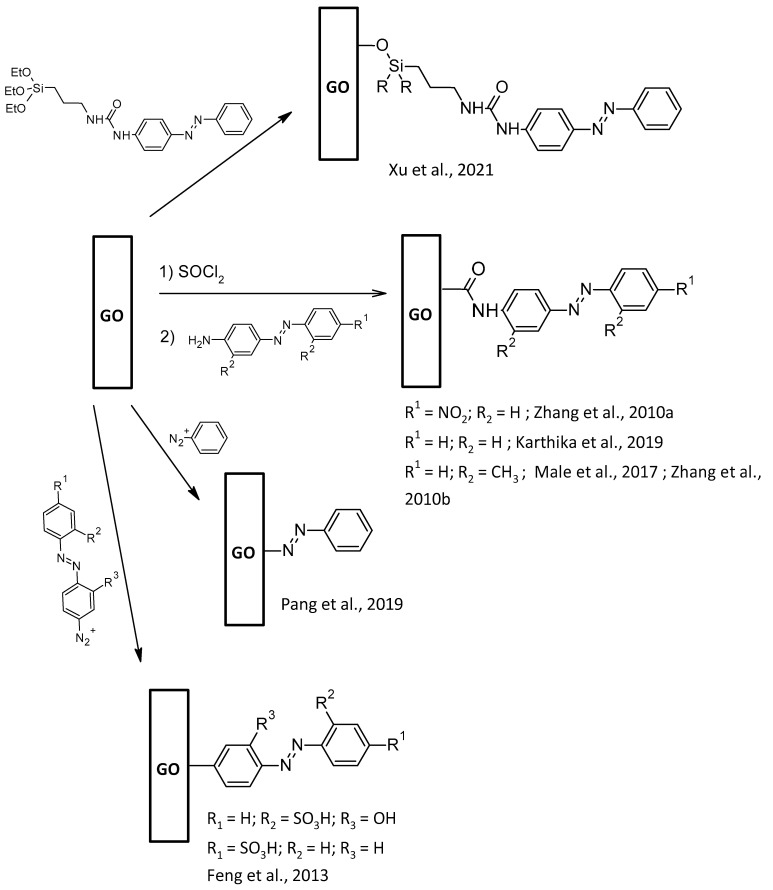
GO-AZO synthetic pathways [93,94,95,96,97,98,99].

AZO chromophore has received extensive research as a molecular solar thermal storage material because of its ability to absorb light in the ultraviolet-visible range and release heat based on reversible isomerization [92]. AZO pre-formed molecules can bond on GO due to an amide linkage (Figure 7). This can lead to strong and stable bonded compounds. Indeed, after treating GO with thionyl chloride, the carboxyl groups are transformed into reactive acyl chloride moieties, and an aniline-based AZO can lead to a hybridized material with a nitro function [93], a nude AZO [94] or a dimethyl-AZO [95,96]. So, this way of functionalization supports electron-donating or electron-withdrawing elements. Diazonium salt can also directly bond the GO to form the AZO moiety in situ (Figure 7), leading after isomerization to a weak C-H···π non-bonding interaction [97]. However, pre-formed AZO diazonium salt can be bonded to GO thanks to the good leaving properties of this function [98]. GO can undergo a Si-O-C bond from 4-(3-Triethoxysilylpropyl-ureido) AZO, even if the FT-IR characterization shows a mixture of the hydrolysis of the Si-OEt groups to Si-OH and further condensation to the Si-O-Si polysilsesquioxane networks of AZO-GO hybrids [99].

**Figure 8:**
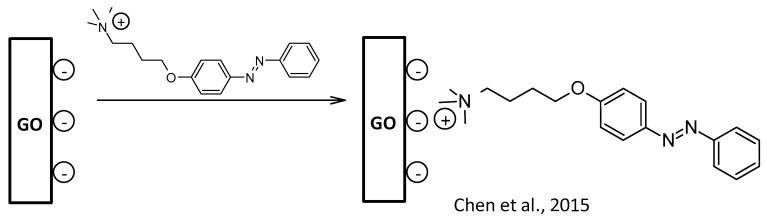
Electrostatic functionalization of GO [100].

Electrostatic interactions between GOscis, a negatively charged material (carboxylate groups) on basal planes and edges (Figure 8), and the azobenzenic ammonium salt occur during the synthesis of cationic AZOs. This led to an interesting azobenzene-surfactant-modified graphene hybrid [100].

Li et al. [101] described a method for producing functionalized graphene using non-covalent stacking interactions. BP2T molecules are used for non-covalent functionalization, and the resulting graphene has superior ammonia-sensing capacities, with a sensitivity three times greater than that of pristine graphene, which is in good agreement with the binding energies derived from the Langmuir isotherm model. These findings provide direct evidence of gas species interactions with graphene functional groups, and the non-covalent approach can be used in various gas detection applications. Shangguan et al. [102] demonstrated the synthesis of AZO-functionalized GNRs utilizing a variety of spectroscopic characterizations. The GNR-AZOs are extremely soluble in common organic solvents, allowing for simple spin coating of films with uniform morphology. The UV-vis-NIR spectra of GNR-AZOs can be modulated quickly and reversibly by alternating UV and visible light irradiation. After at least ten irradiation cycles, such modulation can be repeated without obvious attenuation of the absorption intensity, indicating the photo-fatigue-resistant capability of GNR-AZOs.

## 3. Applications of AZO-Functionalized GO

### Sensors for Depollution

**Figure 9:**
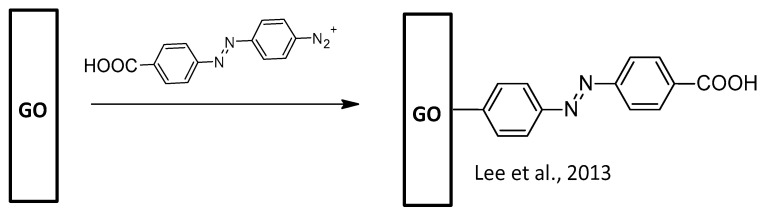
Covalent bonding of AZO on GO [104].

Most AZO-functionalized GO is mainly described for energy storage; very few investigations used the capability of AZO-GO for pollutant sensing and removal. The pesticide residues and metabolites in food, water, and soil are major issues. Fenitrothion (FT) (O, O-dimethyl O-(4-nitro-m-tolyl) phosphorothioate) and modifying graphite pencil electrodes with AZO-GO displays excellent performance for the detection of these pollutants [103]. The lowest LOD (lowest detection limit) for FT can be obtained with RGO/DPA/PGE electrode using square wave voltammetry (SWV, 3.48 nM), while PANI/CGE gives a LOD of 7.20 nM with adsorptive stripping voltammetry (AdSV), and the other references go up to 0.8 µM including SWV. The PGE (pencil graphite electrode) was immersed in a solution containing the AZO-DPA dye (poly(E))-1-(4-((4-(phenylamino)phenyl)diazenyl)phenyl)ethanone), and DPA was electropolymerized over PGE surface. The graphene modification was then applied over the DPA-modified PGE by electro-polymerization in GO solution with various cycles. The determination of FT was performed on a real sample of tomato spiked with different FT concentrations. The recovery of the measured samples ranged between 96.4% and 106.9%, indicating that the proposed method can effectively be applied to determine FT in tomato samples. Another example concerns the use of metal-organic framework-functionalized GO nanocomposites and the reversible detection of high explosives [104]. After preparing the RGO, the AZO moiety was covalently bonded to the GO to form the diazonium salt (as leaving group) under sonication (Figure 9). This AZO-functionalized graphene reacted with a 4,4-stilbene dicarboxylic acid and zinc nitrate to form the sensor. The detection of the explosive vapors (TNT and DNT) was performed by fluorescence quenching.

**Figure 10:**
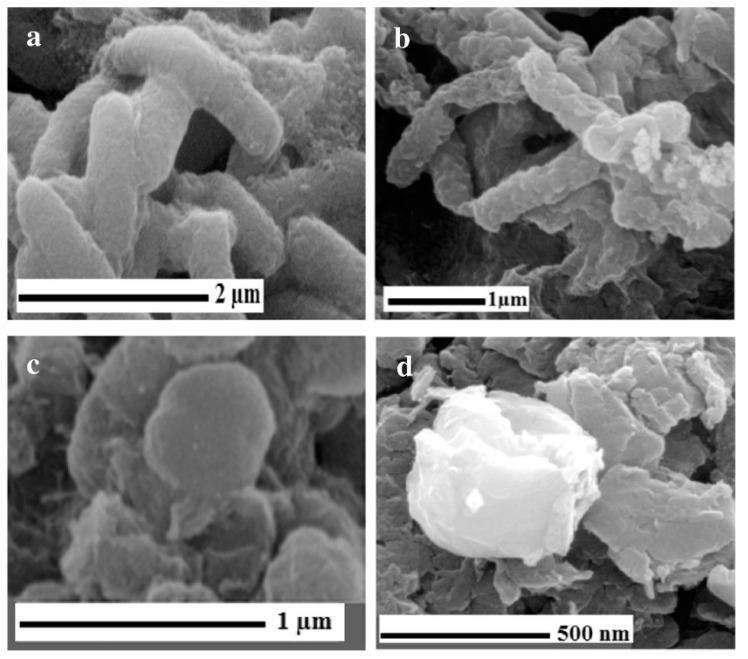
(**a**) SEM images of *E. coli* after incubation with saline solution for 2 h without graphene-based materials, (**b**) *E. coli* cells after incubation with GO-L_2_ dispersion (40 μg/mL) for 2 h, (**c**) *S. aureus* after incubation with saline solution for 2 h without graphene-based materials, and (**d**) *S. aureus* cells after incubation with GO-L_2_ dispersion (40 μg/mL) for 2 h [106].

The removal of bacteria from water is also of great interest, as the WHO declared in 2017 that ESKAPEE bacteria pose a serious public health problem [105]. In this regard, GO/RGO hybrids formed with Schiff bases of AZO pyridinium salt and chromene segments were caused by π-π interactions between GO/RGO and chromene part ligands in addition to electrostatic interactions. As shown in Figure 10, the functionalized material provokes the destruction of the bacterial cell wall and the discharge of Escherichia coli and Staphylococcus aureus cell content [106].

A variety of photo energy conversion or storage devices may be made using photo-induced variations in microstructures, electrical characteristics, steric effects, and optical response of AZO moieties. AZO moieties functionalized with GO/RGO may reflect, prolong, and boost the optically controlled conductance, absorption, catalytic behaviour, and electrostatic response of the composite’s constituents. AZO-GO composites have many advantages: high quantum yields, charge transfer in nanoseconds at the interface, energy storage in chemical bonds, electrochemical catalytic activity, the regulated electrostatic environment surrounding carbon-conjugated structures, and ultrafast isomerization in a few picoseconds (10–12 s) [107]. Table 1 presents some important applications for AZO-GO composites that rely on their unique characteristics.

## 4. Applications of AZO-Gr2Ms Hybrids

### 4.1. Photoswitches

**Figure 11:**
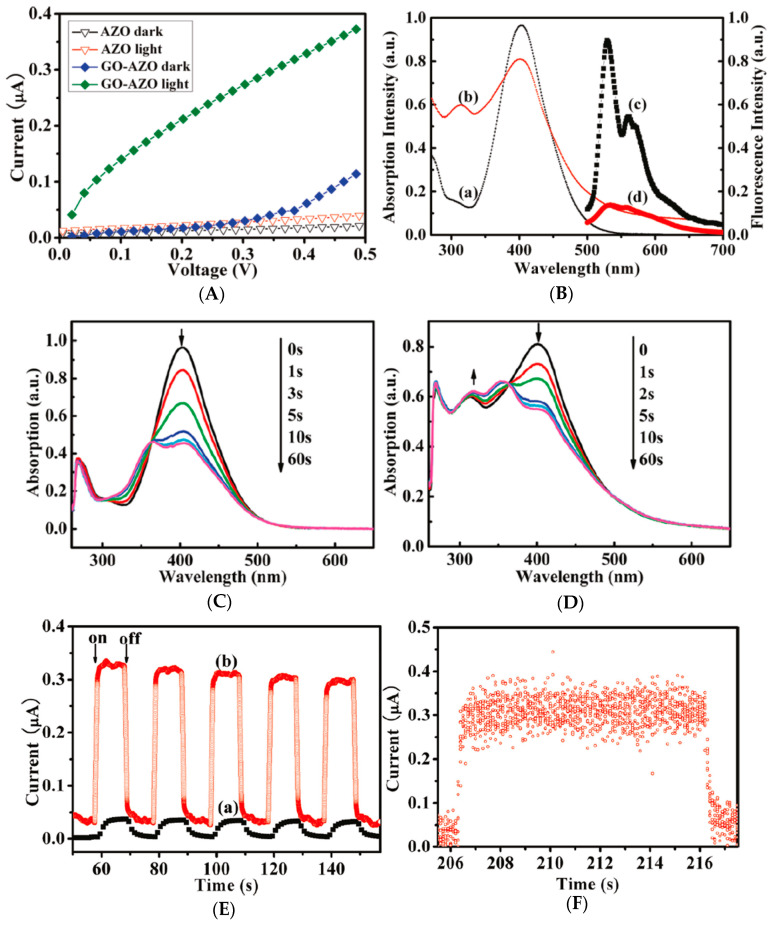
(**A**) Typical *I-V* characteristics of the GO-AZO and pristine AZO without and with UV irradiation (365 nm and 2.45 mWcm^−2^). (**B**) Absorption spectra of (**a**) AZO and (**b**) GO-AZO, and the corresponding fluorescence spectra of (**c**) AZO and (**d**) GO-AZO in anhydrous dimethyl formamide (DMF) at 480 nm. (**C**) Changes in the absorption spectra of AZO and (**D**) GO-AZO in DMF at 365 nm. Arrows refer to the variations due to irradiation. (**E**) Photocurrent response of (**a**) pristine AZO and (**b**) GO-AZO films in 0.1 M KCl aqueous solution at 0.5 V bias, and (**F**) GO-AZO photocurrent responses in single on/off cycles [96].

Photoswitches are molecules that alter structurally when exposed to light irradiation. Depending on the light irradiated wavelength, AZO can stay in two different isomeric forms, such as trans and cis. Its typical isomerization characteristics, including a moderate change in dipole moment, a low photobleaching rate, and a substantial conformational change, make it an excellent choice for the coupling counterpart in light-driven molecular switches. For instance, Kizhisseri et al. [125] found that the combination of AZO and RGO displays phototunable conductance owing to AZO’s light-induced trans-cis isomerization. The non-covalent and covalent functionalization affect the photoconductance characteristics of RGO. Hybrids also exhibit an increased current when exposed to UV light due to the AZO’s trans-cis isomerization. The current of RGO-AZOC2-C (covalent) dwindles substantially after extended UV irradiation because of the steric effect, which limits AZOC2’s photoisomerization capability. In another study, Zhang et al. [96] found that GO-AZO film exhibits excellent reversible photoswitching with a fast response time of <500 ms, a high on/off ratio of 8, and about 800% on/off ratio of photocurrent subjected to UV irradiation to dark. Figure 11 depicts the characteristics of the GO-AZO and pristine AZO under light illumination. Intra-molecular donor-acceptor design with rapid charge transfers makes GO-AZO switch highly sensitive. Depending on the graphene work function of 4.5 eV, the position of the GO conduction band is 4.45 eV. When exposed to UV light, photon penetrates the hybrid material, allowing photoexcited singlet AZO moiety to transfer charge to the GO conduction band, subsequently to the ITO (indium tin oxide) electrode (work function of 4.7 eV). The recombination of nearby electrons and holes immediately reduces the photocurrent after turning off the light, indicating that no hole aggregate occurred in AZO or along the GO-AZO interface. Rapid charge transfer in intra-molecular donor-acceptor arrangement increases photocurrent, making it suitable for UV-modulated photocurrent conversion devices. The photoswitching behavior of AZO derivatives such as 5,6-dihydrodibenzo diazocine was reported [126]. They found that blue light at 370–400 nm can transfer cis to trans with more than 90% efficiency, while green light at 480–550 nm can switch trans back to cis with approximately 100% efficiency. A photoconversion yield of more than 90% is not possible with AZO. However, the trans isomer has higher n-π* absorption than the cis isomer.

### 4.2. Solar Thermal Storage

**Figure 12:**
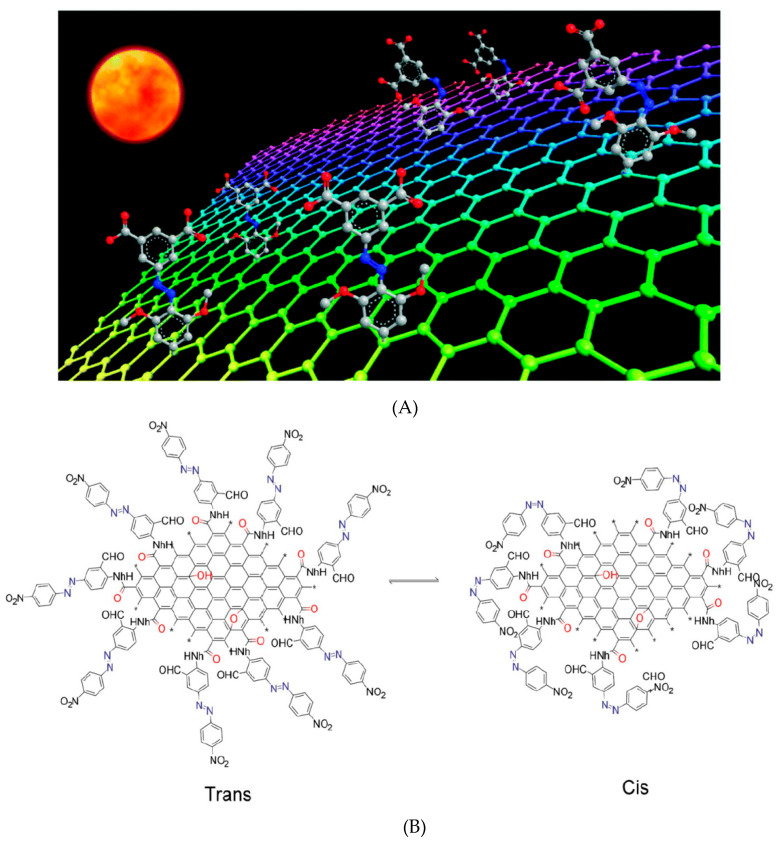
(**A**) Photo-induced isomerization-based AZO-RGO solar thermal storage material: photochemically induced trans-to-cis transition stores solar energy in chemical bonds and releases it as heat tuned by cis-to-trans reversion [111]. (**B**) Cis-to-trans transition of AZO-GO [93,107].

One of the key difficulties is developing multifunctional materials to harness solar energy as a sustainable resource. Solar thermal fuels have the potential to store solar energy in chemical bonds or structures and then release that energy as heat. AZO derivatives have a high potential for solar energy storage owing to their reversible isomerization, high light absorption, and thermal reversion regulated by steric structure and functional group. Feng et al. [108] developed solar thermal storage using the RGO-AZO hybrid and found that Ortho- or para-AZO substitution increases thermal storage (*H*) and cis-to-trans thermal barrier (*E_a_*). RGO-cis-ortho-AZO remains thermally stabilized for a prolonged half-life (5400 h) through intramolecular hydrogen bonds, substantially longer than RGO-para-AZO (116 h). The RGO-para-AZO has a thermal storage capacity of 269.8 kJ kg^−1^ with a single intermolecular hydrogen bond, while RGO-ortho-AZO has a thermal storage capacity of 149.6 kJ kg^−1^ (44.55% less) with several inter- and intra-molecular hydrogen-bonds of AZO. The AZO-GO hybrid has the potential to use as energy storage material with the good thermal stability of cis-hybrid and substantial energy density of around 240 Wh kg^−1^ because of the lightweight structure and significant grafting density of AZO moieties, as stated by Pang et al. [97]. They demonstrate how the C-H…π bonding interacts between AZO and GO in the cis-isomer and decreases the heat shield of π-π* transition, resulting in the outstanding cis-hybrid’s thermal stability. Thus, apart from traditional H-bonds, the weak non-bonding (i.e., C-H…π) interaction plays an essential role in regulating the kinetic and thermodynamic parameters of the AZO-GO. Chromophore RGO-bis-AZO is a photo-isomerizable for high energy density, thermally-stable energy storage material, as demonstrated by Feng et al. [110]. Due to substantial steric hindrance, cis-isomer has a half-life of 1320 h in the dark and is thermally stable, and blue light and heat enhance the reversion followed by solar heat release. The RGO-bis-AZO offers excellent 50-cycle cycling stability with high energy and maximum power densities of 80 Wh kg^−1^ and 2230 Wkg^−1^, respectively. Optimizing molecular interactions with high-density, adjustable RGO-bis-AZO storage allows for the creation of solar-heat conversion materials. Luo et al. [111] reported that AZO-RGO has a considerable energy density of 138 Wh kg^−1^ due to steric hindrance, intermolecular H-bonds, extended storage lifespan (52 days), and an outstanding cycling performance for 50 visible light-irradiated cycles at 520 nm, making substituted AZO-based graphene a stable, high-energy, and recyclable molecular solar thermal storage material. Figure 12A,B illustrate photo-induced isomerization-based AZO-RGO molecular solar thermal storage material and cis-to-trans transition of AZO-GO, respectively. Thus, it can be inferred that high-performing solar thermal fuels derived from AZO-GO/RGO may be improved by raising the grafting density and intramolecular H-bonding interactions.

### 4.3. Memory

**Figure 13:**
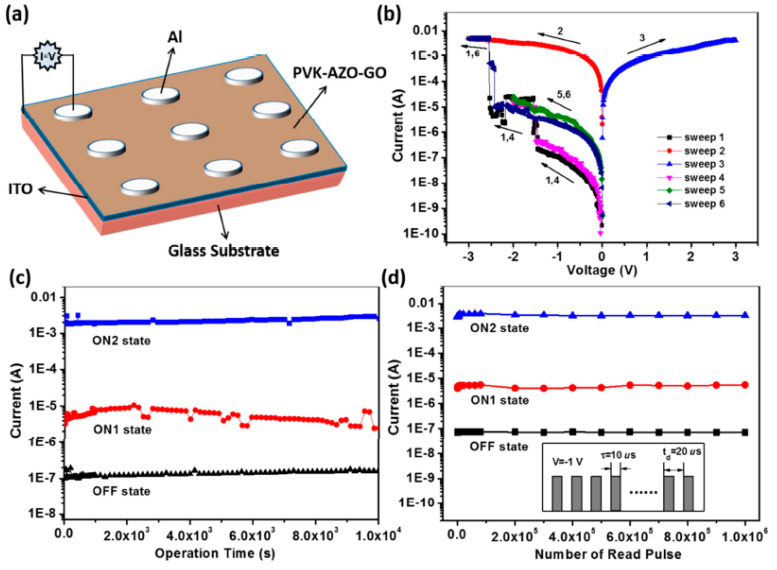
(**a**) Al/PVK-AZO-GO/ITO device: (**b**) *I-V* properties, (**c**) Impact of operating duration on OFF, ON1, and ON2 state currents subjected to −1 V, and (**d**) Impact of reading pulses of −1 V on OFF, ON1, and ON2 state currents. The inset of (**d**) displays the measurement pulse [120].

Due to their inherent conductivity and extraordinarily high specific area (2600 m^2^ g^−1^), AZO-GO materials have the potential to be used in electrochemical capacitors [127]. In addition, its storage capacity enables data to be written and erased in photon mode with great temporal and spatial precision. Min et al. [128] used an all-solution-processed technique to make a non-volatile molecular memory device with an AZO layer between two RGO films as electrodes. The RGO/ABC10 SAM/RGO device demonstrates a reliable nonvolatile memory ability, as shown by the fast-current response to trans-cis isomerization (voltage-controlled). The memory performance remains stable for more than 400 cycles of WRER (write-read-erase-read), and reading, writing, and erasing voltages were −1, 3, and −3 V, respectively. The WRER function was retained after six months of storage, displaying critical features for application in nonvolatile memory and consistent endurance and retention of ON/OFF. The memory device exhibits clear non-destructive and ON/OFF modes across >20,000 readouts for 10,000 s. Additionally, such flexible device shows excellent memory performance under bending stress. Zhang et al. [120] have shown that nonvolatile resistive random access memory (RRAM) with digital-type current switching and high ON/OFF ratio characteristics may significantly increase polymer memory device storage capacity. A poly(N-vinylcarbazole)-functionalized GO with AZO chromophore (PVK-AZO-GO) was synthesized, where carbazole moiety, GO, and AZO function as electron donor (D), electron acceptor (A), and charge trap (T), respectively. The Al/PVK-AZO-GO/ITO device structure and its current-voltage (I-V), and effect of operation time and read pulse is shown in Figure 13. This device shows a nonvolatile ternary WORM memory performance as a result of interaction of field-induced charge-transfer between GO (A) and carbazole moiety (D), as well as later charge trapping at AZO chromophores (T). The obtained OFF: ON1: ON2 current ratio was 1:101.6:104.5, with low switching threshold voltages of −1.53 (ON1) and −2.50 V. (ON2).

### 4.4. Other Applications

**Figure 14:**
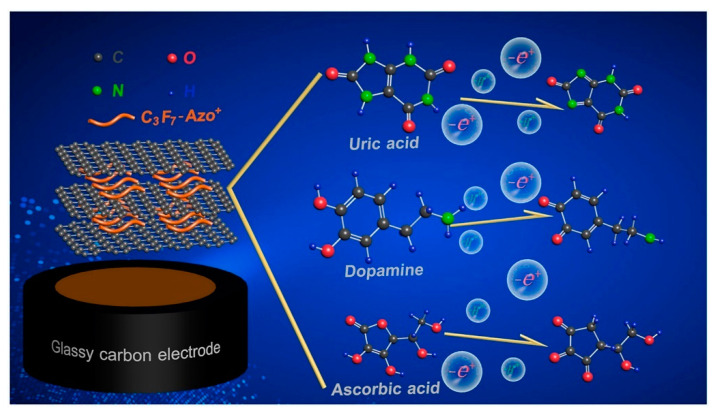
Cationic polyfluorinated AZO-RGO for simultaneous detection of uric acid, dopamine, and ascorbic acid [112].

In addition to applications in solar energy storage, photoswitches, and memory, photochromic carbon nanomaterials with AZO has potential in other emerging fields. Florio et al. [129] built the first graphene multi-layer nano-strain gauge to measure mechanical forces associated with light-driven processes, such as developing polymer surface relief gratings. When irradiated with a light interference pattern, photosensitive film deforms as per spatial intensity fluctuation, resulting in the production of periodic topographies, including surface relief gratings, with the internal pressure surpassing 1 GPa due to grating formation. Deka et al. [123] used AZO nanocluster to create two functionalized graphene composites: one with RGO via π-π stacking and another directly immobilized on the GO. The electrical characteristics of AZO-RGO exhibit n-type behavior, whereas GO shows p-type behavior. Ultimately, when AZO-RGO and GO are coated on a 1 cm  ×  1  cm filter paper substrate to create a junction, it effectively displays the typical diode’s characteristic curve, demonstrating the use of this material in electrical devices. Wu et al. [112] revealed that the C3F7(cationic polyfluorinated)-AZO+/RGO-modified electrode (C3F7-AZO+/RGO/GCE) might determine dopamine (DA), ascorbic acid (AA), and uric acid (UA), simultaneously (see Figure 14), with high anti-interference and stability. The detecting levels are 65 nM, 11 nM, and 8 nM for DA, UA, and AA, ranging from 57.28 to 134.28 μM, 9.23 to 23.45 μM, and 0.04 to 6.01 μM, respectively. Moreover, because of their unique physicochemical features, including large surface area, high adsorption capacity, chemical stability, and recyclability, GO-based nanomaterials, and their composites are regarded as potential adsorbents for removing dyes, heavy metals, and other harmful pollutants from aquatic environments [130].

### 4.5. AZO-Gr2Ms Polymers Composites

**Figure 15:**
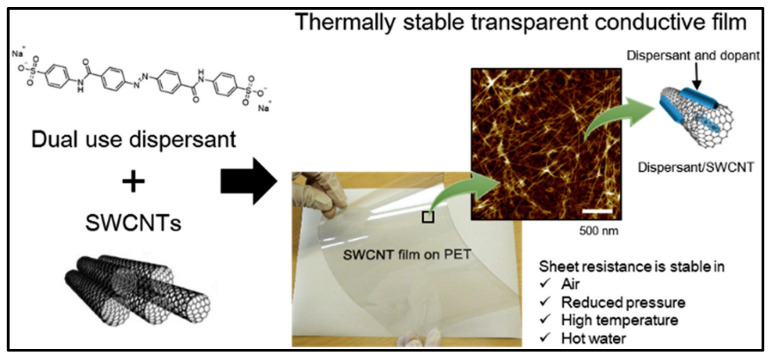
Dual use of anionic AZO derivative as dispersant and dopant for carbon nanotubes for enhancing the thermal stability of transparent conductive films [144].

Non-conjugated AZO groups are those in which two or more AZO groups are linked together using non-conjugated flexible linkers [131,132]. Non-conjugated multi-AZO groups can be introduced in series in polymers [132]. In recent years, there has been much interest in AZO polymers with photoswitchable glass transition temperatures and reversible solid-to-liquid transitions [133]. AZO polymers have been widely investigated as photochromic (AZO) compounds. Light-induced AZO chromophores may create a large and steady in-plane anisotropy, nonlinear optical responses, and imprinting surface-relief structures [134,135,136,137]. The modifications observed in the properties of functional composites are due to the photoisomerization of AZO by the interaction between molecules or groups, considering various photonic and electronic applications [132,138]. Recent studies revealed that a molecular mode with AZO is covalently bonded to carbon nanostructures (graphene or carbon nanotubes), which have the potential for high-density solar thermal storage. Materials with isomerization of single, double bonds (C-C, C=C, and N=N), or conversion [139,140] have widely been studied owing to their special characteristics of reversing the solar energy to be stored in the chemical bonds [141,142]. With the absorption of photons at a particular wavelength, a photoisomerizable molecule can endure structural changes with the transition to a high-energy metastable state. By applying external factors (e.g., heat, light, voltage, or chemical reaction), molecules in the metastable state can return to a stable condition by crossing the thermal barrier. GDs functionalized in AZO-polymer complexes show photo modulation and solar thermal storage capabilities in producing a moiety [143,144,145]. Jintoku et al. [144] demonstrated the air and thermally stable carbon nanotube (CNT)-based transparent conductive material prepared in a one-step process using a simple wet-coating method with an anionic AZO derivative and sulfonate group (Figure 15). The AZO derived from the substituent size is thermally less susceptible, decreasing the thermal desorption of AZO from the CNT surface and further supporting the stability of transparent conductive films at high temperatures, as shown in Figure 15 [144]. Stability refers to the strong intermolecular interactions between the AZO derivative and CNT. The two-dimensional structures of GDs offer a good platform for assembling AZO molecules in a close-packed order, enabling high-density grafting, strong intermolecular interactions, and steric hindrance, which are suitable for controlling the steric configurations and functional groups in composite systems [146].

## 5. Conclusions

This review article summarized the structure, characteristics, synthesis, and applications of AZO-GO/RGO hybrids. Combining Gr2Ms (GO/RGO) with AZO molecules can meet ever-growing and -challenging multifunctional system demands. Oxygen-containing groups on GO/RGO enable covalent and non-covalent composite synthesis, producing well-defined and customizable physical and chemical characteristics. These characteristics include increased quantum effects, effective charge transfer at the interface, electrostatic changes surrounding π-conjugated structures, optically controlled conductance, heat storage in chemical bonds, and steric conformation. These materials can be used in various devices such as photoswitches, photodetectors, phototherapy, molecular junctions, sensors, flexible photonics and electronics, solar thermal storage, smart devices, and biological-based recognition. In addition, these solar-powered materials might lead to eco-friendly, innovative, and sustainable goods. However, there are still some challenges, such as ensuring effective linkages between GO/RGO and AZO moieties, a comprehensive understanding of complex interactions between GO/RGO and AZO, low quantum and thermal storage yields of photoswitching isomers, modulating photochromic and electronic properties to enhance light sensitivity and shorten response time need to address for applying such multifunctional responsive materials with great success. A detailed analysis of the working process of GD-AZO composite materials, focusing on the molecular interaction between these structures, is also required to increase our understanding for future advancement.

## Figures and Tables

**Table 1:** Synthesis, properties, and applications of AZO-grafted GO composites.

Materials	Syntheses	Properties	Applications	References
RGO-AZO	Diazotization	Enhance thermal storage and trans reversion by H-bonds via para- or ortho-replacement of AZO; RGO-para-AZO has a high thermal storage density of 269.8 kJ kg^−1^.	Solar thermal storage	[108]
AZO-GO	Covalent functionalization	Reversible photoisomerization at 300–400 nm, good thermal stability, and high energy density of 240 Wh kg^−1^.	Solar thermal storage	[97]
PCL-RGO-AZO	In situ ring-opening polymerization	High conductivity, conductivity rises by UV irradiation and recovers by visible light irradiation.	Photo switches and reversible optical storage	[109]
RGO-bis-AZO	Covalent grafting	High energy and maximum power densities of about 80 Wh kg^−1^ and 2230 W kg^−1^, respectively.	Solar thermal storage	[110]
AZO-RGO	Covalent grafting	The high energy density of 138 Wh kg^−1^, a 52-day long storage lifespan, and 50-cycle cycling stability at 520 nm.	Solar thermal storage	[111]
AZO-surfactant-modified-GO	Electrostatic interactions	Light-induced reversible photoresponsivity assembly and disassembly.	Photoresponsive supercapacitors	[100]
AZO-RGO-GCE	Exfoliation and restacking	Excellent stability and anti-interference capability.	Determination of ascorbic acid, dopamine, and uric acid	[112]
AZO-GO	Amide linkage	Reversible photoisomerization.	Optic and photonic devices	[93]
AZO-GO-PU	Covalent grafting of the amide linkage	Improved thermal properties and high-water repellence.	Anti-biofouling, fluid transportation, sensors, self-cleanings, super-hydrophobic valves, battery, and fuel cell	[94]
PANI-RGO-AZO	Covalent grafting, functionalization, and aniline polymerization	A specific capacitance of 328 F g^−1^, 80% capacitance retention after 1500 continuous charge-discharge cycles, and high electrochemical performance.	Supercapacitors	[95]
PANI-GO-AZO	Diazotization	High reversibility and specific capacitance retention after 500 cycles, with a capacitance of 478.3 F g^−1^. Excellent photosensitive electrochemical properties under UV irradiation and capacitance change rate of 52.57%.	Photoresponsive supercapacitor	[113]
GO-AZO	Covalent grafting	Rapid trans-cis photoisomerization and increased reversible photoswitching with fast response time <500 ms and a high on/off ratio of 8.	Photoswitching	[96]
AZO-GO-PVA	Covalent grafting	Mimic the reversible grabbing-release motions of a claw upon UV/visible irradiation.	Smart devices	[99]
AAZO-GO-PEG	Covalent grafting	High absorbance under visible light illumination, high latent heat of 84.5 J g^−1^, and photothermal conversion efficiency of 91%.	Solar thermal storage	[114]
AZO-RGO	Covalent grafting	High solar thermal energy storage density of 112 Wh kg^−1^ with 32-day prolonged storage. Outstanding cycling stability for 50 visible light-irradiated cycles, suggesting more than 4.5 years of use.	Solar thermal storage	[115]
RGO-bisAZO-2	Covalent grafting	High power density (2517 W kg^−1^), high energy density (131 Wh kg^−1^), good cycling performance (50 cycles), and prolonged half-life (37 days).	Photothermal fuels	[116]
tri-AZO-RGO	Covalent grafting	High power density (3036.9 W kg^−1^), high energy density (150.3 Wh kg^−1^), and extended half-life (1250 h). Film releases 23.6–69.7% of stored heat, raising the temperature by 2–7 °C.	Photothermal energy	[117]
AZO-RGO-GNP	Electrostatic interactions	Photochemical behavior du to Gemini AZO-surfactant stabilizers and electrochemical performance governed by light irradiation.	Optic and photonic devices	[118]
AZO-GO	Esterification reaction	Good photosensitive electrochemical properties (high energy and maximum power densities of 47 Wh Kg^−1^ and 156.6 W Kg^−1^, respectively).	Solar thermal fuels and energy storage devices	[119]
PVK-AZO-GO	Amidation reaction and covalent grafting	A ternary electrical switching and nonvolatile WORM (write-once-read-many-times) memory performance, with low switching threshold voltages of −1.53 (ON1) and −2.50 V (ON2) and an OFF: ON1: ON2 current ratio of 1: 10^1.6^:10^4.5^.	Multilevel memory devices	[120]
AFGO-AZO-PI	Covalent polyimide	Improved response time (i.e., 0.5 ms) with transmission loss of 0.167 dB/cm.	Photoswitches	[121]
PolyAZO (Bismarck brown Y)-RGO	Non-covalent π-π stacking	Good repeatability in chemiresistor response per regeneration cycle and resistance is sensitive to O_2_ concentration.	Chemiresistor for mitochondrial consumption	[122]
AZO nanocluster-RGO, AZO nanocluster-GO	Non-covalent π-π stacking and direct immobilization	RGO-AZO nanocluster functions as n-type while GO p-type.	p-type diode and n-type diode	[123]
AZO-BNB-t8-RGO	Non-covalent π-π stacking	Improved linear optical absorption, high nonlinear optical absorption, and saturable absorption coefficient.	Nonlinear optical material	[124]
AZO-RGO	Covalent and non-covalent	Phototunable conductance with light-induced AZO trans-cis isomerization. Non-covalent functionalization provides better photoconductance tuning than the covalent counterpart, which may constrain the AZO photo-isomerization activity.	Molecular electronics	[125]

RGO-AZO: reduced graphene oxide-azobenzene, PCL: poly(ε-caprolactone), GCE: glassy carbon electrode, PU: polyurethane, PANI: polyaniline, PVA: poly(vinyl alcohol), AAZO: amino AZO, PEG: polyethylene glycol, GNP: gold nanoparticle, PVK: poly(N-vinyl carbazole), AFGO: amino functionalized GO, and PI: polyimide.
